# Current Management of Non-ST-Segment Elevation Acute Coronary Syndrome

**DOI:** 10.3390/biomedicines12081736

**Published:** 2024-08-02

**Authors:** Pablo Díez-Villanueva, César Jiménez-Méndez, Pedro Cepas-Guillén, Andrea Arenas-Loriente, Ignacio Fernández-Herrero, Héctor García-Pardo, Felipe Díez-Delhoyo

**Affiliations:** 1 Cardiology Department, Hospital Universitario La Princesa, 28006 Madrid, Spain; 2 Cardiology Department, Hospital Universitario Puerta del Mar, 11009 Cádiz, Spain; cesarjm91@gmail.com; 3 Cardiology Department, Hospital Clinic, 08036 Barcelona, Spain; pedro.cepasguillen@gmail.com (P.C.-G.); arelor95@gmail.com (A.A.-L.); 4 Cardiology Department, Hospital Universitario Doce de Octubre, 28041 Madrid, Spain; nacho.7693@gmail.com (I.F.-H.); felipediezdelhoyo@hotmail.com (F.D.-D.); 5 Cardiology Department, Hospital Universitario Río Hortega, 47012 Valladolid, Spain; hgarciapardo@hotmail.com

**Keywords:** acute coronary syndrome, non-ST-elevation coronary syndrome, antiplatelet therapy, revascularization, cardiac rehabilitation, elderly

## Abstract

Cardiovascular disease constitutes the leading cause of morbimortality worldwide. Non-ST-segment elevation acute coronary syndrome (NSTE-ACS) is a common cardiovascular condition, closely related to the ageing population and significantly affecting survival and quality of life. The management of NSTE-ACS requires specific diagnosis and therapeutic strategies, thus highlighting the importance of a personalized approach, including tailored antithrombotic therapies and regimens, combined with timely invasive management. Moreover, specific and frequent populations in clinical practice, such as the elderly and those with chronic kidney disease, pose unique challenges in the management of NSTE-ACS due to their increased risk of ischemic and hemorrhagic complications. In this scenario, comprehensive management strategies and multidisciplinary care are of great importance. Cardiac rehabilitation and optimal management of cardiovascular risk factors are essential elements of secondary prevention since they significantly improve prognosis. This review highlights the need for a personalized approach in the management of NSTE-ACS, especially in vulnerable populations, and emphasizes the importance of precise antithrombotic management together with tailored revascularization strategies, as well as the role of cardiac rehabilitation in NSTE-ACS patients.

## 1. Introduction

Cardiovascular (CV) disease represents the leading cause of mortality and morbidity worldwide, with acute coronary syndromes (ACS) constituting the most frequent manifestation of incident CV disease [1], including an estimated 5.8 million new cases of ischemic heart disease in Europe in 2019 [1]. Due to the progressive aging of our societies, the incidence of ACS, particularly non-ST-segment elevation ACS (NSTE-ACS), is increasing, especially in older patients [2]. 

The current definition of NSTE-ACS includes both unstable angina (UA) and non-ST-elevation myocardial infarction (MI). MI is defined as a combination of criteria, namely an increase or decrease in a cardiac biomarker (high-sensitivity troponin, hs-cTn, with at least one value over 99th percentile) and one of the following: symptoms of myocardial ischemia, electrocardiogram (EKG) findings suggesting ischemia, new Q waves on EKG, new regional wall motion abnormality compatible with ischemic etiology in any CV imaging technique or finding of intracoronary thrombus detected on angiography or autopsy [3].

Traditional and emergent CV risk factors interact in the pathophysiology of ACS. The underlying mechanisms of ACS involve a complex interplay of atherosclerosis, endothelial dysfunction, oxidative stress, inflammation, and platelet activation, resulting in the rupture or erosion of the atherosclerotic plaque within coronary arteries. Less common mechanisms include coronary spam, spontaneous coronary artery dissection, or coronary embolism [4].

Appropriate management of ACS is vital for enabling rapid risk stratification and tailored therapy, ensuring guideline-adherent care to reduce both mortality and morbidity and to optimize healthcare resources. The advent of potent antithrombotic drugs offers a significant opportunity in the management of NSTE-ACS patients. Additionally, invasive treatment and percutaneous coronary revascularization significantly improve prognosis in these patients [2]. Moreover, integrating cardiac rehabilitation into the management plan yields substantial benefits [2]. This personalized approach is essential, especially in vulnerable populations, such as the elderly, patients with chronic kidney disease, or frail patients, due to their unique physiological challenges and increased susceptibility to complications, ultimately improving outcomes and enhancing their quality of life. In this review, we summarize the current management of NSTE-ACS in clinical practice, specifically addressing the role of antiplatelet therapy and the invasive approach, also highlighting the importance of cardiac rehabilitation and secondary prevention to improve the prognosis of our patients.

## 2. The Role of Antiplatelet Treatment in NSTE-ACS

Antithrombotic therapy is the cornerstone of treatment strategies for patients presenting with NSTE-ACS [5]. There have been no major changes in the type of antithrombotic treatments in recent years. However, the timing of administration, duration, and combination of antiplatelet agents remain subjects of debate [6]. Indeed, dual antiplatelet therapy (DAPT) consisting of aspirin (ASA) combined with a P2Y12 inhibitor (P2Y12i), such as prasugrel, ticagrelor, or clopidogrel, plays a key role in the management of ACS due to its significant reduction in ischemic events despite an increased risk of bleeding [7]. The 2023 European Society of Cardiology (ESC) guidelines recommend the use of a potent P2Y12i (ticagrelor or prasugrel) agent over clopidogrel due to its superiority in reducing ischemic events [8,9]. Among these agents, prasugrel should be considered more preferential to ticagrelor for patients undergoing percutaneous coronary intervention (PCI). This recommendation is supported by the results of the ISAR-REACT 5 trial, which observed a significant reduction in death, myocardial infarction, and stroke without an increase in bleeding complications in a prasugrel group [10]. However, two limitations of the study should be addressed: its open-label design with a small sample size and its intention-to-treat analysis with 32.5% of patients in the ticagrelor group and 30.4% in the prasugrel group not being treated with the assigned drug. Finally, clopidogrel should only be used when prasugrel or ticagrelor are contraindicated or in patients at a high bleeding risk (e.g., need for anticoagulation) [11]. Intravenous antiplatelet drugs include novel P2Y12i agents (such as cangrelor) and glycoprotein (GP) IIb/IIIa inhibitors (such as eptifibatide and tirofiban). Cangrelor has proven to be effective in preventing intra- and post-procedural stent thrombosis in P2Y12i-naïve patients, and it may be considered in selected patients undergoing PCI, particularly those who are unable to receive oral drugs [2,12]. GP IIb/IIIa inhibitors should be considered if there is evidence of no reflow or a thrombotic complication during PCI [2,13].

**Figure 1:**
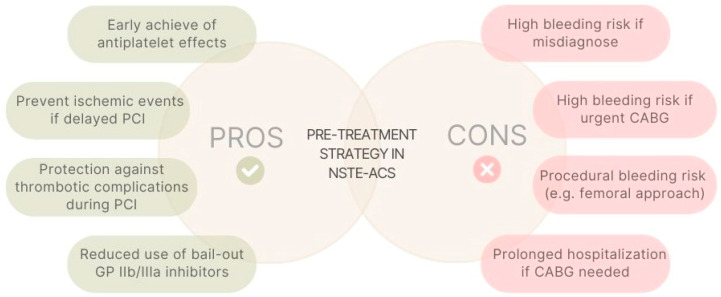
Potential advantages and disadvantages of pre-treatment strategy in NSTE-ACS. PCI, percutaneous coronary intervention. CABG, coronary artery bypass grafting. GP IIb/IIIA, glycoprotein IIb/IIIA.

A current key issue is the timing of P2Y12i administration. The routine pre-treatment with a P2Y12i in NSTE-ACS patients in whom coronary anatomy is not known and early PCI (<24 h) is planned is no longer recommended [2]. This recommendation is supported by the ACCOAST trial, which failed to demonstrate a reduction in ischemic events at 30 days after a prasugrel loading dose prior to (median of 4.3 h) versus at the time of PCI, but also showed a significantly increased bleeding risk [14]. A pre-treatment strategy could have a theoretical advantage in helping achieve an early anti-platelet effect since oral drugs have a relatively slow onset of action. However, misdiagnosis and bleeding risk, as well a prolonging hospitalization if coronary artery bypass graft is performed, can tip the risk–benefit balance in favor of avoiding pre-treatment [15,16] (Figure 1). Therefore, an assessment of coronary anatomy before the administration of a P2Y12 inhibitor appears to be the most reasonable strategy in an ACS setting, unless invasive management is delayed (>24 h) and the patient’s bleeding risk is low [17].

The recommended default dual antiplatelet therapy (DAPT) regimen after an ACS consists of a P2Y12i plus aspirin for 12 months followed by aspirin monotherapy [8,18]. However, this is an evolving area, and novel paradigm DAPT strategies are being evaluated, aiming to maintain efficacy while improving safety [19]. Multiple randomized clinical trials (RCTs) are testing new antiplatelet regimens and alternative durations of antiplatelet therapy, suggesting the use of a P2Y12i in isolation following a brief period of DAPT (1–3 months) [20,21]. Supported by MASTER-DAPT, STOPDAPT-2, and ULTIMATE-DAPT, P2Y12i monotherapy reduces the risk of bleeding compared with prolonged DAPT [22,23]. In this regard, according to recent recommendations, the use of single antiplatelet therapy (preferably P2Y12i) should be considered in patients with no high ischemic risk and those who are event-free after 3–6 months of DAPT (class IIa and IIb recommendation for 3 months and 6 months DAPT, respectively) [2].

Going further, challenging the fundamental concept of DAPT post-PCI, current investigations in the field are exploring a new paradigm shift by considering total aspirin-free strategies [24]. The STOPDAPT-3 trial is the first randomized controlled trial evaluating an aspirin elimination strategy comparing DAPT with prasugrel and prasugrel monotherapy (3.75 mg daily) in a high-bleeding risk population. Prasugrel monotherapy did not demonstrate superiority over DAPT at the 1-month follow-up for the primary bleeding endpoint, and thrombotic events occurred more frequently in the monotherapy arm [25]. Cautious interpretation of the results and further investigation are needed for aspirin elimination strategies post-PCI. Focusing attention on a more complex population, NEO-MINDSET (percutaneous coronary intervention followed by monotherapy instead of dual antiplatelet therapy in the setting of acute coronary syndromes; NCT04360720) and LEGACY (less bleeding by omitting aspirin in non-ST-segment elevation acute coronary syndrome patients; NCT05125276) trials are currently evaluating the impact of monotherapy in ACS patients undergoing PCI (Table 1). On the other hand, extending DAPT for more than 12 months has demonstrated a reduction in ischemic events at the expense of an increased risk of bleeding [26]. This extended therapy for secondary prevention should be considered in high–moderate ischemic risk patients without a high bleeding risk [27]. 

A not negligible number of patients with ACS also have an indication for long-term anticoagulation, with the consequent high bleeding risk and need for careful management of antiplatelet treatment based on their individual risk [34]. Evidence of management of antiplatelet therapy in patients with an indication for long-term oral anticoagulation (OAC) is derived from subgroups of RCTs. Overall, in patients without contraindications for non-vitamin K antagonist oral anticoagulants (NOACs) (e.g., mechanical prosthetic valves or moderate to severe mitral stenosis), the use of NOACs is preferable due to their reduced bleeding risk [35,36]. As the default strategy, after up to 1 week of triple antithrombotic therapy following the ACS event, dual antithrombotic therapy using a NOAC and a single oral antiplatelet agent for up to 12 months is recommended [2]. Extending triple therapy for longer than 1 week up to 1 month should be considered in high ischemic risk patients or with other anatomical or procedural characteristics that are judged to outweigh the bleeding risk [2,37]. In this scenario, clopidogrel is the antiplatelet agent of choice.

In conclusion, antithrombotic strategies are under continuous development and treatment decisions must be individualized by weighing the antithrombotic benefits against the risk of bleeding. The results of ongoing clinical trials may determine the best antithrombotic approach for patients with NSTE-ACS.

## 3. Revascularization and Timing in NSTE-ACS

### 3.1. Benefits of an Invasive Strategy in Patients with NSTE-ACS

**Figure 2:**
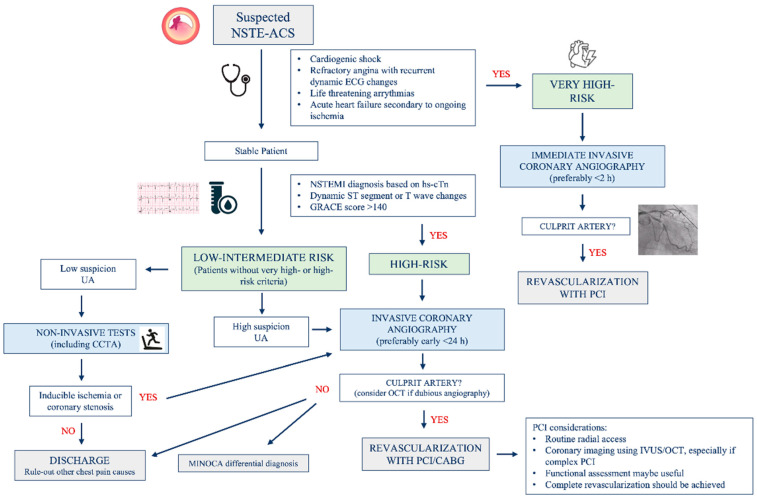
Selection of invasive strategy and revascularization therapies in patients presenting with NSTE-ACS [2]. NSTE-ACS, non-ST-segment elevation acute coronary syndrome; ECG, electrocardiogram; PCI, percutaneous coronary intervention; NSTEMI, non-ST-segment elevation myocardial infarction; GRACE, global registry of acute coronary events; UA, unstable angina; CCTA, coronary computed tomography angiography; OCT, optical coherence tomography; MINOCA, myocardial infarction with non-obstructive coronary arteries; IVUS, intravascular ultrasound.

In the recent two decades, coronary angiography and revascularization (when indicated) have been adopted as the strategy of choice in patients presenting with NSTE-ACS (Figure 2) [2,38]. Routine versus selective invasive strategies have been studied in randomized clinical trials, and their aggregated results have been analyzed in several meta-analyses. Despite similar rates of all-cause mortality, a reduction in the incidence of myocardial infarction and CV death has been consistently found [39]. Importantly, clinical trials comparing conservative and invasive strategies are dated, typically overestimate periprocedural infarction and bleeding rates, and do not integrate modern developments with proven prognostic impact, such as radial access [40], the use of drug-eluting stents (DESs), complete revascularization [41], and novel antiplatelet agents [8,18].

### 3.2. Risk Stratification

Early risk stratification upon admission is essential for a tailored therapeutic strategy in patients with NSTE-ACS because those at a higher risk benefit the most from revascularization [2,40,41,42] (Figure 2). There is a consensus on basing the initial short-term risk stratification on a combination of clinical history, symptoms, vital signs, physical examination, EKG findings, and hs-cTn levels. 

Prompt recognition of very high-risk patients is essential given the therapeutic implications. This group includes patients with unstable presentations (e.g., in cardiogenic shock), with progressive development of heart failure secondary to ongoing ischemia, with refractory angina despite medical treatment (especially if EKG changes), and those with severe ventricular arrhythmias after presentation [2].

Several prognostic scores have been developed to facilitate decision making in stable patients by estimating long-term risk of all-cause mortality and MACE [2]. Among these, the GRACE risk score offers the best discriminative performance and is therefore recommended [2]. In this sense, a dichotomic cut-off value of 140 identifies high-risk subjects amenable for routine invasive management. However, the identification of high-risk patients has been simplified in recent years in CPG. In this sense, the two main initial diagnostic tests, the EKG and the hs-cTn, have prognostic value. Any EKG changes suggestive of acute ischemia imply there is a high risk, which includes dynamic changes in the T wave and ST segment, as well as transient ST-segment elevation. For its part, the diagnosis of NSTEMI according to the current 4th universal definition (rise or fall of hs-cTn above the 99th percentile of healthy individuals) also identifies high-risk patients amenable of initial invasive management [3]. 

Of note, since the widespread adoption of hs-cTn assays, the proportion of high-risk patients has significantly increased, currently representing more than 80% of NSTE-ACS cases [43]. For this reason, a routine invasive strategy is widely recognized as the standard of care (Figure 2). Patients without high-risk features, traditionally referred as UA and currently considered low-intermediate risk patients, may undergo non-invasive tests, including coronary computed tomography angiography, or may be referred for elective straight coronary angiography, depending on clinical suspicion (Figure 2). According to current recommendations, if the clinical suspicion of UA is high, patients should be managed similarly to those with high-risk features [2]. 

### 3.3. Timing of Coronary Angiography

Once the decision to undergo an invasive strategy has been made, it is mandatory to decide the precise timing, an important topic currently under debate [44]. In this sense, an early invasive strategy refers to routine angiography within 24 h of presentation. Patients with very high-risk NSTE-ACS should undergo immediate coronary angiography (and percutaneous coronary intervention if needed), which involves emergency management [2,38,45]. Traditionally, a dichotomist cut-off point of <2 h has been recommended [46], although this extent may vary according to local capacities. Moreover, in patients with high-risk criteria, a routine early invasive strategy has been recommended in guidelines as the standard of care with the highest level of evidence [46]. However, available data merit a more detailed discussion. The TIMACS trial randomized 3031 patients with NSTE-ACS to undergo either routine early intervention or delayed intervention [47]. At 6 months, the primary outcome (a composite of death, myocardial infarction, and stroke) occurred in 9.6% of patients in the early intervention group compared with 11.3% in the delayed intervention group (HR 0.85; 95% confidence interval [CI], 0.68 to 1.06; *p* = 0.15). In the VERDICT trial, 2147 high-risk NSTE-ACS patients were randomized to an invasive coronary angiography within 12 h or standard invasive care within 48 to 72 h [48]. After a median follow-up of 4.3 years, the primary endpoint, a combination of all-cause death, myocardial infarction, refractory myocardial ischemia, or hospital admission for heart failure did not significantly differ between groups (27.5% in the early invasive strategy versus 29.5% in the standard care group; HR 0.92; 95% CI, 0.78–1.08). More recently, the largest meta-analysis to date, including more than 10,000 patients, concluded that early invasive strategies do not reduce MACE in patients with NSTE-ACS [49]. Although a widespread use of an early invasive strategy is associated with shorter hospital stays and fewer recurrent ischemic episodes [49], these findings are not sufficient to improve survival. Accordingly, patients should undergo coronary angiography on an elective, non-emergency basis but should seek a prompt invasive diagnosis, as well as PCI if required, preferably in the first 24 h whenever possible. In the SWEDEHEART registry, early invasive management was achieved in only 35% of the 34,666 patients with NSTE-ACS [43]. In our setting, the results of the IMPACT-TIMING-GO registry showed that an early invasive strategy was performed in only 37.8% of patients [43]. In both real-life registries, the percentage of patients in whom catheterization was performed in the first 72 h of admission was greater than 80%, which probably reflects more realistic times in accordance with the daily management of NSTE-ACS.

### 3.4. Revascularization in Patients with NSTE-ACS

Revascularization of the culprit artery in patients with ACS reduces MACE and therefore should be routinely performed if technically feasible [2,38]. The decision to revascularize should be made on angiographic findings, although the use of intracoronary imaging in doubtful cases, especially with optical coherence tomography, is very useful and thus should be considered. Functional assessment of the culprit artery is strongly contraindicated. 

Multivessel disease is present in up to 50% of ACS patients. In the STEMI context, except in cardiogenic shock, the benefit of complete revascularization has been consistently demonstrated [50] and is strongly recommended [2]. However, there are no dedicated trials comparing complete revascularization against infarct-related artery only PCI in NSTE-ACS patients. The available observational evidence suggests a comparable benefit in terms of survival [41], so a goal of complete revascularization should also be recommended, although with a lower level of evidence, in patients with NSTE-ACS [2,38]. In this context, optimizing PCI outcomes using intracoronary imaging techniques improves prognosis, especially in patients with complex lesions [51]. Finally, a physiological assessment of non-culprit lesions may be helpful in identifying functional significant lesions. However, given the frequent associated MI-related microvascular dysfunction, which can lead to fractional flow reserve (FFR) false negatives, and the presence of functionally non-significant vulnerable plaques, with the potential to become unstable in the short-term, FFR and non-hyperemic indices should be used with caution [52].

No dedicated randomized trials comparing percutaneous vs. surgical revascularization have been published to date in patients with NSTE-ACS [53]. Except for very high-risk patients in whom emergent revascularization, generally by PCI, is mandatory, in hemodynamically stable patients, it is recommended to decide the best revascularization strategy in accordance with the general principles of Clinical Practice Guidelines [2,38]. Globally, around 1–10% of patients undergo CABG during admission [9,43].

In summary, patients with NSTE-ACS should undergo catheterization routinely, preferably in the first 24 h of admission if myocardial infarction or EKG changes. Depending on the severity and complexity of the coronary disease, the best revascularization approach will be performed. High-quality PCI integrating the use of radial access, drug-eluting stents, intracoronary imaging, coronary physiology, and modern antithrombotic treatment is essential to guarantee the best results. In very high-risk patients, generally those with hemodynamic instability, emergent invasive management should be performed in order to treat the culprit lesion percutaneously.

## 4. Considerations in Special Populations

### 4.1. Elderly

Age is linked to a higher CV risk. Notably, elderly patients account for more than one third of ACS cases, with non-ST-elevation myocardial infarction (NSTEMI) being the most common presentation in this population [54]. As the population continues to age, the incidence of ACS among older patients is expected to rise. Consequently, clinical guidelines have included specific recommendations regarding the management of this elderly population. Accordingly, most elderly patients with NSTEMI should undergo invasive coronary angiography [2]. Despite this, older adults have historically been under-represented in the main clinical trials and have been less frequently referred to invasive management [55]. Table 2 summarizes clinical studies comparing invasive versus conservative management in elderly patients with NSTEMI. 

Interestingly, a recent meta-analysis suggests that routine invasive management in older patients with NSTEMI does not reduce the composite of all-cause mortality and myocardial infarction within one year compared to conservative management [60]. However, a sub-analysis of this study indicates that invasive management could reduce the risk of MI or urgent revascularization [60]. Significant limitations should be considered in this meta-analysis, since only six trials with a limited number of patients (1479, nearly one-third from the After Eight study) were included. Moreover, the follow-up was restricted to one year due to insufficient data. Additionally, the median timing of angiography was not documented. 

Undoubtedly, this population faces intrinsic challenges with a high level of comorbidities, such as anemia or chronic kidney disease (CKD). Therefore, some considerations should be made when referring older patients to invasive management (i.e., the radial approach should be prioritized; DES are preferable in all clinical scenarios, even when short dual antiplatelet regimens are intended to be prescribed; and bridging therapies are discouraged in patients receiving anticoagulation) [61].

### 4.2. Frailty

Frailty is a condition defined as a loss of biological reserve leading to impaired responses to stressor events [62]. The prevalence of frailty is higher in older adults, affecting up to 20% of the population over 80 years old, and is even higher in those with CV disease [63]. Frailty significantly and adversely impacts the prognosis in several CV scenarios, such as heart failure or ACS [64,65]. Moreover, frail patients are less frequently referred to invasive management, receive potent antiplatelet inhibitors less often, and have a poorer quality of life [66]. This scenario is also specifically addressed in the current clinical guidelines. Validated clinical scales, such as the Clinical Frailty Scale or FRAIL scale, which are easy to use, are highly recommended [67]. Furthermore, frailty is associated with an increased risk of in-hospital adverse events, functional decline, and prolonged hospital stays [68].

To date, the MOSCA-FRAIL trial is the first trial including elderly (over 70 years old) and frail patients (defined by a score of four or more on the Clinical Frailty Scale) with NSTEMI randomized to either invasive or conservative strategies [69]. The primary endpoint was the number of days alive and out of the hospital during the first year. Crossover was allowed in the case of recurrent ischemia. Notably, invasive treatment in frail older patients did not show survival benefits [59]. More extensive clinical trials are necessary to determine the optimal revascularization strategy in frail patients with NSTEMI, in whom a holistic approach is of great importance.

### 4.3. Chronic Kidney Disease

CKD is characterized by a decreased glomerular filtration rate that persists for over three months [70]. As CKD advances, the risk of CV events, including acute coronary syndrome (ACS), rises significantly. More than 30% of patients with ACS, particularly in the elderly, show some degree of renal insufficiency [71]. Moreover, CKD significantly affects the prognosis of patients, with ACS being associated with as much as a 30% increase in mortality [72].

CKD patients are less frequently referred to invasive management. Furthermore, the use of contrast agents in diagnostic procedures such as angiography can exacerbate kidney damage. While preventing contrast-induced nephropathy is advisable, the evidence on effective prevention strategies is mixed. Generally, hydration is recommended for patients with ACS and CKD who are undergoing coronary angiography [73]. CKD is also associated with a lower prescription of potent antiplatelet medications, probably due to a higher bleeding risk. Antiplatelet agents must be chosen and dosed appropriately to avoid adverse effects [74].

Finally, preventative strategies and secondary prevention are crucial for this high-risk population. Regular monitoring of kidney function, management of risk factors, and early intervention in the progression of CKD can help reduce adverse events [75].

## 5. Management of Cardiovascular Risk Factors and Cardiac Rehabilitation

Preventing CV disease is crucial due to its potentially fatal impact. Estimating CV disease risk remains essential to identify individuals at a high risk of new or recurrent CV events who would benefit most from preventive therapies. In the primary prevention scenario, the use of various scores are recommended, such as the Systematic Coronary Risk Evaluation 2 (SCORE2) or the Atherosclerotic Cardiovascular Disease (ASCVD) risk score, all of which are based on traditional CV risk factors [75]. Additionally, novel CV scores, such as QR4, identify new risk groups (e.g., malignancy survivors or women with gynecological disorders). This score, which is more multifaceted, has been prospectively validated, demonstrating superior performance over other CV scores in the UK population [76]. Notably, both ST-elevation myocardial infarction (STEMI) and NSTEMI share common CV risk factors. Nevertheless, their relative prevalence is different, and patients with NSTEMI are usually older, with a previous history of heart disease and a higher frequency of high blood pressure and lower tobacco consumption [77]. 

Secondary prevention targets are equal in all ACS [2]. Main recommendations regarding secondary prevention controlling CV risk factors are summarized in Table 3 [78,79,80].

Recommended exercise includes from 150 min (ideally >210 min) per week of moderate to high-intensity aerobic training, divided over at least 3 days. Resistance training is also advised with special benefits in older or frail patients, with 2–3 days per week, >20 min sessions, and 10–15 repetitions involving 8–10 muscle groups [81,82] (Table 4). 

Cardiac rehabilitation (CR) stands as a multidisciplinary approach not only to supervised exercise training but also to a holistic educational program that individually encircles other physical, social, emotional, and occupational issues related to CV health [83]. 

**Figure 3:**
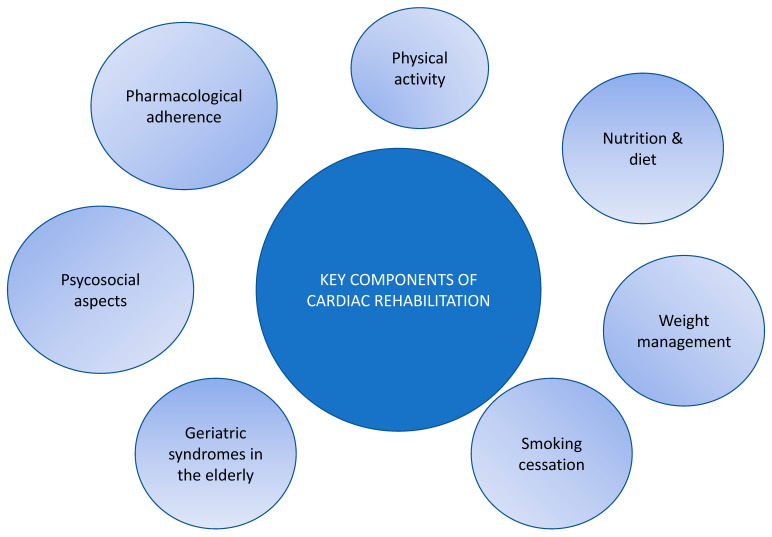
Key components of cardiac rehabilitation.

The complete structure of a CR program includes three phases [84]. Phase I begins right after the coronary event and implies early mobilization and the introduction to general principles of treatment (e.g., complete tobacco cessation). Phase II should begin as soon as possible after discharge (ideally within the first month) or after the first outpatient visit because delays in referral are one of the main causes of lower adherence to these programs. This phase poses as the main part of multidisciplinary work, usually including a cardiac risk assessment, individualized exercise treatment for 2 to 3 months in 24 to 36 sessions, nutritional formation, smoking cessation assistance, and psychosocial management or occupational counselling (Figure 3). Phase III is focused on the maintenance of lifestyle changes and patient self-care.

CR is highly recommended after ACS to reduce deaths, hospital readmissions, and improve the quality of life in NSTEMI according to AHA/ACC (class IB) and ESC guidelines (IA) [38,46]. The management and benefits of CR do not seem to differ between STEMI or NSTEMI [46] but, as NSTEMI patients tend to be older, problems associated with age like cognitive impairment, malnutrition, sarcopenia, frailty, or polypharmacy may require a specific assessment [2,85]. Despite proven benefits, referral rates to CR programs remain low. Different factors have been identified, such as female sex, older age, or low sociocultural environment. Other individual factors include the patient’s perception of lack of efficacy, difficulties of transport to the CR center, or comorbidities. Health system issues with a low number of CR programs available or professionals’ lack of awareness of treatment benefit also contribute to the fact than only one quarter of the eligible CR patients are finally referred to the programs [86]. The benefit of CR can be greater in the frail and comorbid elderly than in the younger or more robust elder patients [87]. However, the exercise program should firstly address mobility, balance, and resistance training over aerobic training, and an educational part focused on multimorbidity self-care rather than single-disease approach could be useful [88]. Regarding women, improvements in functional capacity and quality of life are equivalent to their male counterparts [79]. Flexible hours and days, women-only CR programs, or alternative forms of CR delivery have been studied, with positive results being recorded [89].

**Figure 4:**
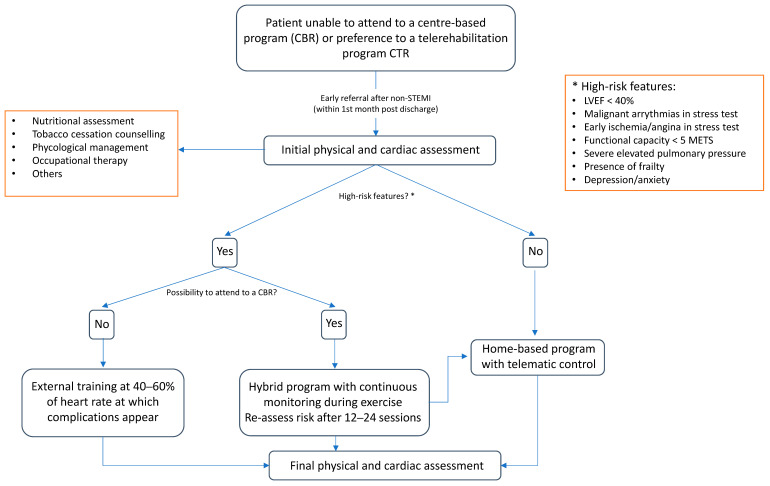
Proposed model for a phase II rehabilitation program flowchart.

In this scenario, cardiac telerehabilitation (CTR) can be an upcoming alternative to center-based cardiac rehabilitation (CBCR) to overcome referral barriers [90]. There are several studies that show both safety and cost effectiveness, even implementing high-intensity interval training. However, there are several drawbacks to these programs. The structure of an optimal program is still uncertain as most studies are small and their methodology is variable. The ideal candidate profile for CTR is not well established [91]. Several factors have been related to a lower adherence to CTR, including older age, lower educational level, lower exercise capacity, and coronary artery bypass grafting [90,91]. Importantly in CTR, the patient’s or at least family’s knowledge on technology systems must be adequate. Patients at a high risk of events after the initial assessment (e.g., ventricular arrhythmias or induced ischemia in stress test, persistent angina, or low ventricular ejection fraction) might need cardiac monitoring during exercise, thus leading to the recommendation of a CBCR program. An intermediate approach to this problem could be hybrid programs, which can include an initial part of CBCR followed by a CTR once the risk of adverse events is proven to be low (Figure 4). Initial experiences show similar short-term benefits compared to CBCR [92] and, along with CTR, they could help to improve patients’ adherence to CR programs. Nevertheless, and similarly to CTR, the optimal structure of these programs is yet to be elucidated, with there being a lack of long-term evidence [93].

## 6. Conclusions

The management of NST-ACS patients still represents a clinical challenge. Careful selection of appropriate antithrombotic therapies combined with optimal timing and strategies for revascularization, following current recommendations, improve prognosis. A personalized approach should be adopted in vulnerable populations, such as frail patients or patients with CKD. Referral to CR programs and the early implementation of secondary prevention measures should be warranted. 

## Figures and Tables

**Table 1:** Randomized controlled trials investigating aspirin-free strategies after percutaneous coronary intervention (PCI) over recent years.

	SMART-CHOICE [28]	GLOBAL LEADERS [29]	TWILIGHT [30]	TICO [20]	STOPDAPT-2 [31]	STOPDAPT-2 ACS [32]	STOPDAPT-3 [25]	T-PASS [33]	ULTIMATE-DAPT [23]
Year	2019	2019	2020	2020	2021	2021	2023	2023	2024
Population	N = 2993	N = 15,968	N = 7119	N = 3056	N = 3045	N = 4169	N = 5966	N = 2850	N = 3400
ACS (%)	58%	47%	65%	100%	38%	100%	75%	100%	100%
STEMI (%)	18%	28%	0%	36%	49%	79%	57%	40%	28%
Treatment strategy	DAPT 3 M	DAPT 1 M	DAPT 3M	DAPT 3 M	DAPT 1 M	DAPT 1–2 M	-	DAPT < 1 M	DAPT 1 M
	Clopidogrel vs. DAPT	Ticagrelor vs. DAPT	Ticagrelor vs. DAPT	Ticagrelor vs. DAPT	Clopidogrel vs. DAPT	Clopidogrel vs. DAPT	Prasugrel vs. DAPT	Ticagrelor vs. DAPT	Ticagrelor vs. SAPT
Cardiovascular endpoints	✓ MACCE	✘ MACCE	✓ MACCE	✓ NACE = MACE	✓ NACE ✓ MACE	✘ NACE ✘ MACE	✓ MACCE	✓ NACE = MACCE	✓ MACCE
Safety endpoints	✓ BARC 2–5	= BARC 3–5	✓ BARC 2–5	✓ TIMI major	✓ BARC 3–5	✓ BARC 3–5	✘ BARC 3–5	✓ BARC 3–5	✓ BARC 2–5

Acute coronary syndromes (ACS), ST-segment elevation myocardial infarction (STEMI), dual antiplatelet therapy (DAPT), single antiplatelet therapy (SAPT). Cardiovascular endpoints are measured by net adverse clinical event(s) (NACE), major adverse cardiovascular event(s) (MACE), and major adverse cardiovascular or cerebrovascular event(s) (MACCE). Safety endpoints are measured by Bleeding Academic Research Consortium (BARC) and Thrombolysis In Myocardial Infarction (TIMI). M: Month(s). Symbols in cardiovascular and safety endpoints denote: “✓” significant difference, “✘” failure to reach the endpoint, “=” no significant difference between study arms.

**Table 2:** Clinical trials including elderly non-ST-elevation acute coronary syndrome patients.

Study	Population	Age, Sex	Results
After Eighty [56]	457 patients	84.8 years 50.5% women	Primary outcome (myocardial infarction, urgent revascularization, stroke, and death): 40.6% invasive group vs. 61.4% conservative group; *p* = 0.0001
Italian Elderly ACS [57]	313 patients	81.8 years 50% women	Primary outcome (myocardial infarction, CV rehospitalization, disabling stroke, severe bleeding, and death): 27.9% invasive group vs. 34.6% conservative group; *p* = 0.26
RINCAL [58]	251 patients	85.0 years 50% women	Primary outcome (non-fatal myocardial infarction, and death): 18.5% invasive group vs. 22.2% conservative group; *p* = 0.39
MOSCA-FRAIL [59]	167 patients	85.5 years 52.5% women	Primary outcome (days alive and out of the hospital): 284 days in invasive group vs. 312 days in conservative group; *p* = 0.12

Abbreviations: CV, cardiovascular; ACS, acute coronary syndrome.

**Table 3:** Management of traditional cardiovascular risk factors in non-ST-elevation acute coronary syndrome patients [2].

Risk Factor	Recommendations
Hypertension	Target blood pressure < 140/80 mmHg or even <130 mmHg if tolerated. If frail or very older (over 80 years): lenient control
Dyslipidemia	Target LDL-cholesterol ≤ 55 mg/dL and >50% baseline reduction in very high cardiovascular risk patients.
Diabetes	Target glycated hemoglobin level of 7–7.5%. Frail or terminal ill patients: avoid hypoglycemia, lenient control.
Diet	Mediterranean diet adherence
Smoke	Smoke cessation
Obesity	Avoid obesity. Overweight may be permitted based on clinical profile.

**Table 4:** Recommendations for prescription of physical exercise [80,82]. All recommendations should be based on patient’s needs and preferences to enhance exercise adherence.

	Aerobic	Resistance	Other
Frequency	3 or more days per week (ideally 6–7 days per week)	2–3 sessions per week. Recommend 48 h between sessions.	Flexibility training, balance training and inspiratory muscle training can be considered according to patient’s comorbidities, disabilities, or special conditions
Intensity	40–59% of peak oxygen consumption 40–59% heart rate reserve 12–14/20 Borg scale	30–70% of the 1-RM for the upper body and 40–80% of the 1-RM for the lower body with 12–15 repetitions	
Time	>20 min/session (ideally 45–60 min)	>20 min per session	
Volume	150 min to >210 min per week	1–3 sets of 12–15 repetitions for muscle group	
